# Rate of Cardiovascular Implantable Electronic Device-Related Infection at a Tertiary Hospital in Saudi Arabia: A Retrospective Cohort Study

**DOI:** 10.7759/cureus.27078

**Published:** 2022-07-20

**Authors:** Rashed Khubrani, Abdullah S Alghamdi, Abdulrahman A Alsubaie, Thamer Alenazi, Abdulkreem Almutairi, Faris Alsunaydi

**Affiliations:** 1 College of Medicine, King Saud Bin Abdulaziz University for Health Sciences, Riyadh, SAU; 2 Internal Medicine/Infectious Diseases, King Abdulaziz Medical City, Riyadh, SAU

**Keywords:** staphylococcus, local infections, systemic infections, device-related infections, infection, pacemaker, device, cied infection, cardiac implantable electronic device (cied)

## Abstract

Introduction

Cardiovascular implantable electronic devices (CIEDs) are long-term cardiac treatments that address a variety of cardiac diseases. In the recent years, a steady growth has been noticed in CIEDs, mainly due to expanding indications for their usage. Possible device-related infection, whether pocket or systemic, which leads to high morbidity and mortality, is one of the most worrying complications. In addition, there are limited studies conducted on the topic of CIED infection rate and their clinical presentation both regionally and locally.

Methods

In this retrospective cohort study, we reviewed the medical records of all patients with CIEDs who presented to our medical center (implanted, followed up, or referred to our hospital) between January 2016 and January 2019.The medical records were extracted from the BestCare electronic medical records system (ezCaretech Co, Seoul, Korea). All consecutive patients were included as we had no exclusion criteria.

Results

During the three years of the study period, a total of 612 patients with CIEDs were identified at our medical center. Among this cohort, 436 subjects (71.2%) were male and 176 (28.8%) were female. Thirty-four patients experienced device-related infections from among the total patient population (n = 612) who presented with CIEDs between January 2016 and January 2019, for a total rate of 5.6%. Of the infected patients, 29 (85%) presented with local infections and five (15%) presented with systemic infections.

Conclusion

The infection rate of 5.6% observed in this study was higher than expected. Therefore, we conclude that action should be taken to reduce infection rates at our medical center to at least that seen in prior studies or below that, if possible. Moreover, we found that CIED infections were often caused by *Staphylococcus* species and commonly affected the elderly and patients with chronic diseases such as diabetes and hypertension. Most of the identified cases were local infections, although systemic infections were common in those with renal disease. Further studies are needed to control the risk factors and to better understand the role of antibiotics, antiseptic prophylaxis, and other methods in avoiding CIED infection and associated complications.

## Introduction

Cardiovascular implantable electronic devices (CIEDs) are long-term cardiac treatments that address a variety of cardiac diseases. CIEDs include a range of devices, such as implantable cardioverter defibrillators (ICDs) for tachyarrhythmia management, pacemakers for bradyarrhythmia treatment, biventricular pacemakers providing cardiac resynchronization therapy with or without a defibrillator, and implantable loop recorders [1-4].

CIEDs provide patients with cardiac disease with an improved quality of life and increased longevity [4]. Patients undergoing CIED therapy are most commonly elderly or have significant comorbidities, placing them at higher risk of developing complications [5,6]. One of the most serious complications of CIEDs is infection, which carries a significant risk of morbidity and mortality in this vulnerable population [4]. Clinical presentation and device management differ significantly depending on the site of infection, the severity of the infection, and the clinical features of the patient [4].

CIED infections are predominantly categorized in two main classes: pocket infections and systemic infections [2,7]. CIED infections occur as a consequence of contamination at the time of the implantation or replacement [5]. Rates of infection vary significantly, ranging from 0.5-1% at the first implantation (representing a narrower range of variation) and from 1-7% at device replacement (representing a wide range of variation) [1,4].

In most cases, complete device removal is necessary in addition to intravenous antibiotics [1,6,8]. In addition, due to previous study findings, most guidelines recommend removing the generator and all leads even if the infection is only limited to the pocket. This recommendation also includes abandoned leads and all foreign material. For example, in a previous study, 71.4% of patients with retained material showed recurrence [7].

CIEDs are lifesaving for patients suffering from a wide range of cardiac diseases. However, no study has been conducted on this topic in Saudi Arabia to date. Hence, this research is of critical importance in informing tailored medical and health policy interventions. We note that a multidisciplinary approach involving infectious disease specialists, cardiologists, and cardiothoracic surgical teams is often required to improve outcomes in patients with CIED infections [4].

In this study, we aimed to identify the rates of infection and to describe patients’ clinical presentations, profile causative pathogens, and determine the occurrence of comorbidities in order to provide critically important information on the epidemiology of CIED infections with the ultimate goal of facilitating appropriate interventions.

## Materials and methods

This is a retrospective cohort study in which the medical records of all patients with CIEDs who presented to our medical center (implanted, followed up, or referred to our hospital) between January 2016 and January 2019 were reviewed. The medical records were housed using the BestCare electronic medical records system (ezCaretech Co, Seoul, Korea). All consecutively presenting patients were included as we had no exclusion criteria.

This study was performed at the King Abdulaziz Cardiac Center (KACC) located within the King Abdulaziz Medical City (KAMC) complex (which includes the National Guard Hospital) in Riyadh, Saudi Arabia.

Definition

In this study, CIED-related infections were categorized into two groups: pocket infections and systemic infections. Clinical diagnoses of CIED infections can be determined through local signs of pocket inflammation, which may appear as erythema, wound discharge, erosion, swelling, pocket warmth, and/or pocket pain, or by detecting signs of systemic infections (such as fever and chills).

CIED infections were confirmed based on positive cultures from the pocket device, blood, and leads [9,10,11]. The accepted definition of CIED-related infection also includes cases of negative cultures with local inflammatory signs, as well as suspected infections on clinical judgment [12].

Data collection process

A request was sent to the medical records database at KAMC/KACC to obtain the medical record-based number of patients who presented with CIEDs between January 2016 and January 2019. The data was collected from the hospital case files by our team members using the BestCare system. We abstracted data on rates of infection as well as information on clinical presentation, comorbidities, and causative organisms.

Data analysis

Study data were entered into a Microsoft Excel spreadsheet (Microsoft Corporation, Redmond, Washington, United States), and IBM SPSS Statistics for Windows, Version 26.0 (Released 2019; IBM Corp., Armond, New York, United States) was used for data management and analysis. Descriptive statistics were calculated to describe the distribution of medical and demographic variables. Continuous variables were presented as means and standard deviations (SDs), while categorical variables were presented as numbers and percentages. T-tests and Chi-square tests were used to compare numerical and categorical data across groups. A p-value of <0.05 was considered the threshold for statistical significance.

Ethical considerations

The study obtained approval from the Institutional Review Board (IRB) of King Abdullah International Medical Research Center, Riyadh, Saudi Arabia (approval number 1277/19). Patient confidentiality was ensured and data was used only for study purposes. Access to the data was restricted to research faculty and staff. The confidentiality of all patients was preserved and no names or medical record numbers were included within the study dataset. Data was stored on password-protected computers in locked rooms. The requirement for informed consent was waived by the IRB due to the retrospective nature of our study. This work was conducted in accordance with the principles of the Declaration of Helsinki and its later amendments.

## Results

During the three years of the study period, a total of 612 patients with CIEDs were identified. Among this cohort, 436 subjects (71.2%) were male and 176 (28.8%) were female. Thirty-four patients experienced device-related infections from among the total patient population (n = 612) who presented with CIEDs between January 2016 and January 2019, for a total rate of 5.6%. Of the infected patients, 29 (85%) presented with local infections and five (15%) presented with systemic infections.

Body mass index (BMI) at baseline was used to categorize patients into the following groups according to WHO standards: underweight (< 18.5 kg/m2), n = 25 (8.5%); normal weight (18.5-24.9 kg/m2), n = 175 (28.6%); overweight (25-29.9 kg/m2), n = 171 (27.9%); and obese (≥ 30 kg/m2), n = 214 (35%); the mean BMI was 28.4 kg/m2, with a standard deviation of 8.1.

We calculated descriptive statistics for baseline demographic and medical variables and compared patient characteristics between the infected (n = 34, 5.6%) and non-infected (n = 587, 94.4%) groups, as summarized in Table 1.

**Table 1:** Baseline characteristics of 612 consecutively presenting patients with CIEDs BMI, body mass index; SD, standard deviation; CIED, cardiac implantable electronic devices

Variable		Non-Infected Group	Infected group	p-value
Age (mean ± SD )		59.54 ± 23,11	57,32 ± 22.19	.865
Gender	Male (n, %)	(71.8%)415	21 (61.8%)	.319
	Female (n, %)	163(28.2%)	13 (38.2%)	.209
BMI (mean ± SD)		28.33 ± 8,10	27.86 ± 7,51	.803

In general, patients who undergo the CIED procedure are clinically unwell and typically have several comorbidities. Certain medications also increase the risk of infection. A total of 253 of the enrolled patients (41.3%) were taking anticoagulants, while 14 (2.3%) were taking immunosuppressive medications. In our study, we found that hypertension and diabetes mellitus were the most commonly occurring comorbidities, with 409 (66.8%) and 368 (60%) affected patients, respectively. A range of other comorbidities, including cardiomyopathy (n = 287, 46.9%), heart failure (n = 247, 40.4%), coronary artery disease (n = 182, 29.7%), chronic kidney disease (n = 127, 20.8%), myocardial infarction (n = 122, 19.9%), coronary artery bypass graft (n = 87, 14.2%), valvular surgery (n = 63, 10.3%), chronic obstructive pulmonary disease (n = 47, 7.7%), cancer (n = 40, 6.5%), skin disorders (n = 33, 5.3%), and congenital heart defects (n = 10, 1.6%), were found in this population as well. We found no statistically significant differences between infected and non-infected patients for any of the evaluated comorbidities, as shown in Table 2.

**Table 2:** Comorbidities in patients with CIEDs (n = 612) CABG, coronary artery bypass graft; CAD, coronary artery disease; CHD, congenital heart defect; CIED, cardiac implantable electronic devices; CKD, chronic kidney disease; COPD, chronic obstructive pulmonary disease

Comorbidities	Non-Infected Group (n, %)	Infected group (n, %)	p-value
Hypertension	388 (67.1%)	21 (61 %)	.519
Diabetes	347 (60 %)	21 (61%)	.841
Cardiomyopathy	271 (46.9%)	16(47%)	.984
Heart Failure	236 (40.8 %)	11 (32.4%)	.328
CAD	168 (29.1%)	14 (41.2%)	.133
CKD	121 (20.9%)	6 (17%)	.646
Myocardial Infarction	115 (19.9)	7 (20%)	.922
CABG	83 (14.4 %)	4 (11.8%)	.736
Valvular Surgery	58 (10 %)	5 (14.7%)	.384
COPD	47 (8.1%)	0 (00%)	.084
Cancer	40 (6.9%)	0 (0,0%)	.113
Skin Disorder	32 (5.4%)	1 (2.9 %)	.536
CHD	9 (1.6%)	1 (2.9 %)	.536

In regard to clinical presentation, fever was the most commonly presenting symptom (n = 17, 50%). Other findings among patients with CIED infections included chills (n = 12, 35%), pocket pain (n = 12, 35%), pus discharge (n = 9, 26.5%), pocket swelling (n = 9, 26.5%), pocket erythema (n = 7, 20%) skin erosion (17%), and pocket warmth (n = 2, 5.9%).

**Figure 1:**
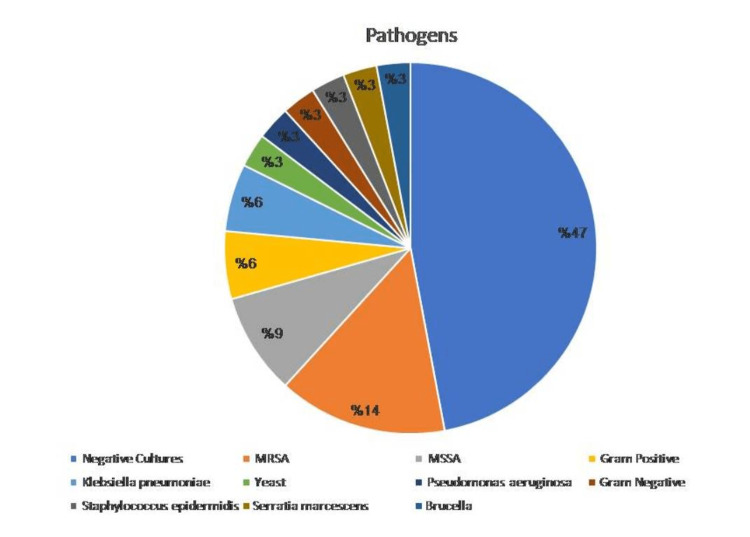
Pathogens isolated from patients with CIED infections MRSA, methicillin-resistant *Staphylococcus aureus*; MSSA: methicillin-sensitive *Staphylococcus aureus*; CIED: cardiac implantable electronic device

With reference to determining which bacterial species were responsible for these infections, we found that the majority of infected patients (n = 18, 52.94%) had positive cultures according to the microbiology report; 16 (47.05%) of the patients showed no growth in their cultures. The pathogen distribution is shown in Figure 1.

The causative microorganisms for these infections included methicillin-resistant *Staphylococcus aureus* (MRSA) in five of the patients and methicillin-sensitive *Staphylococcus aureus* (MSSA) in three of the patients. Two patients had Gram-positive cocci and two had *Klebsiella pneumoniae*. Lastly, yeast, *Brucella *spp., Gram-negative bacilli, *Staphylococcus epidermidis*, *Serratia marcescens*, and *Pseudomonas* species were reported in one patient each

The median time interval between device implantation and the approximate onset date of infection was 56 days (interquartile range = 366 days (A3); minimum duration, six days; maximum duration, 969 days; mean duration, 228 days.

## Discussion

CIED implantation as well as rates of infections have been increasing rapidly over the past decade, and the rate of infections has been increasing disproportionately to the increased rate of CIED implantation [13,14,15]. A regional study conducted by Sadeghi et al. at the Kerman University of Medical Sciences in Iran (March 2011 to March 2015, 3,205 patients) identified device infections in 85 patients (2.7%) with a mean age of 62 ± 16 years (range, 16-92 years) [16].

In addition, research on the rate of CIED-related infections conducted by Prutkin et al. found an implantable cardioverter defibrillator-related infection rate of 1.7% [17]. Another study conducted by Tarakji et al. at the Heart and Vascular Institute at the Cleveland Clinic (Cleveland, Ohio, United States) detected a 0.5% rate of cardiac implantable device infections for primary implants and a much higher (1-7%) rate for secondary interventions [18]. In this study, the rate of infection was determined to be 5.6%, representing a very high rate compared with prior studies [16-18]. This may be explained by factors including the age distribution of the enrolled patients, as well as distribution of comorbidities and the number of referred patients. Approximately 64.7% of the infected patients were 60 years of age or older, and more than 70% of the enrolled patients had one or more coexisting illnesses.

Moreover, according to the findings of Voigt et al., CIED infections in the United States had risen by 12% between 2004 and 2006. The study demonstrated that the number of CIED implantations continued to increase after 2003, from 199,516 in 2004 to 222,940 in 2006, representing a 12% increment. However, in the same period, the number of CIED infections increased from 8,273 in 2004 to 12,979 in 2006, representing a 57% increment [14].

The increase in the rate of CIED-related infections has prompted the American Heart Association (AHA) to develop a scientific statement addressing this concern [1]. In the United States, the number of patients with CIED-related infections continues to rise faster than the rate of implantation [19]. For example, it is well known that patients with diabetes are more prone to infections as compared with those without diabetes. One important cause of the proclivity towards infections detected within the present study may be deficient immunity in patients with diabetes [20]. Another study conducted by Ayman et al. found that, between 2000 and 2011, 816 consecutively presenting patients with confirmed CIED infections who were profiled in this study had a range of comorbid conditions including hypertension (53.1%), coronary disease (53.1%), clinical heart failure (48.2%), atrial fibrillation (44.3%), diabetes mellitus (31.9%), and end-stage renal disease (7.9%) [21].

Another factor associated with infection risk that is relevant to the present study is that nine (26%) of the infected patients evaluated in the current study were taking anticoagulants. This relatively high rate is in line with a previous meta-analysis that found that oral anticoagulant use was statistically significantly associated with an increased rate of CIED infections [12]. Moreover, we detected an association between chronic kidney disease and systemic infection, as 60% of the patients with systemic infections had chronic kidney disease (p = .007). In general, renal insufficiency has been shown to dramatically increase the risk of infections complicating pacemaker implantation or ICD surgery [6,17,22]. However, renal dysfunction has shown the strongest risk association with CIED infections [1].

None of our patients who experienced symptoms of pocket pain showed systemic infections. Therefore, device-related pain seems to be indicative of local infection based on the evidence available to date, although systematic infections should not be ruled out in the differential diagnosis of infectious symptomology. In general, local infections are more common than systemic infections; these infections are accompanied by symptomologies such as edema and localized pain [7,11]. It is of critical importance to recognize that the extent of infection may be underestimated in patients presenting with a localized pocket infection [2], and that systemic infection may be missed in the differential diagnosis. This is critically important as patients with systemic infections have prolonged hospital stays and a higher mortality rate than those with local infections, and timely interventions dramatically alter the course of the disease. However, although some studies conducted in Italy, the United States, and Japan have reported a trend towards a higher incidence of systemic infections in patients with CIED implants, local infections continue to comprise more than half of patients with CIED-related infections in these study populations [23-25].

All of our positive patients’ cultures showed monomicrobial growth, and none of the infections were polymicrobial. In contrast, a previous study showed that, although monomicrobial infectious continued to represent the vast majority of cases (78%), a small proportion of the enrolled patients (10%) had polymicrobial infections [2]. We also note that *Staphylococcus* species accounted for half of the identified pathogens (n = 9, 50%) in the present study and that these causative organisms have been reported as the major causative microbiological agents for CIED infections in prior research [1,2,26].

We also note that, despite clear evidence of clinical CIED infection in the majority of the culture results, we found some unclear culture results as well. For instance, one culture showed Gram-positive cocci and Gram-negative bacilli, without specific pathogens, and negative cultures were obtained in approximately 47% of the cases. This might be a result of starting antibiotic medication before cultures are obtained in many of our cases, or because culture samples may need to be incubated for a longer period of time in order to obtain reliable findings (or a mix of these and other factors) [21].

This cohort of patients with CIED-related infections represents a unique investigation regionally (i.e., no other studies have examined this topic in Saudi Arabia). This is a significant strength of the current study, which was likewise highly powered and rigorously designed. Nevertheless, a number of important limitations must be considered in interpreting the results of this research. First, the rate of referrals to tertiary care centers for patients with suspected CIED-related infections has been increasing in recent years, and as such a bias may exist concerning the detected rates of infection when comparing across study populations. Moreover, few patients received antibiotic therapy before presenting to our hospital, thus potentially confounding the results of microbiology testing. Lastly, this was a single-center study and, therefore, the generalizability of the results is limited.

## Conclusions

The infection rate of 5.6% observed in this study was higher than expected. Therefore, we conclude that action should be taken to reduce infection rates at our medical center to that seen in prior studies or below that, if possible. Moreover, we found that CIED infections were often caused by *Staphylococcus* species and commonly affected the elderly and patients with chronic diseases, such as diabetes and hypertension. Most of the identified cases were local infections, although systemic infections were common in those with renal disease. Further studies are needed to control the risk factors and to better understand the role of antibiotics, antiseptic prophylaxis, and other methods in avoiding CIED infections and associated complications .

## References

[1] Baddour LM, Epstein AE, Erickson CC (2010). Update on cardiovascular implantable electronic device infections and their management: a scientific statement from the American Heart Association. Circulation.

[2] Tarakji KG, Chan EJ, Cantillon DJ (2010). Cardiac implantable electronic device infections: presentation, management, and patient outcomes. Heart Rhythm.

[3] Baddour LM (2010). Cardiac device infection--or not. Circulation.

[4] Lambert CT, Tarakji KG (2017). Cardiac implantable electronic device infection. Cleve Clin J Med.

[5] DeSimone DC, Sohail MR (2018). Approach to diagnosis of cardiovascular implantable-electronic-device infection. J Clin Microbiol.

[6] Pichlmaier M, Knigina L, Kutschka I (2011). Complete removal as a routine treatment for any cardiovascular implantable electronic device-associated infection. J Thorac Cardiovasc Surg.

[7] Ahmed FZ, James J, Cunnington C (2015). Early diagnosis of cardiac implantable electronic device generator pocket infection using ¹⁸F-FDG-PET/CT. Eur Heart J Cardiovasc Imaging.

[8] Da Costa A, Kirkorian G, Cucherat M (1998). Antibiotic prophylaxis for permanent pacemaker implantation: a meta-analysis. Circulation.

[9] Chamis AL, Peterson GE, Cabell CH (2001). Staphylococcus aureus bacteremia in patients with permanent pacemakers or implantable cardioverter-defibrillators. Circulation.

[10] Chua JD, Wilkoff BL, Lee I, Juratli N, Longworth DL, Gordon SM (2000). Diagnosis and management of infections involving implantable electrophysiologic cardiac devices. Ann Intern Med.

[11] Sohail MR, Uslan DZ, Khan AH (2007). Management and outcome of permanent pacemaker and implantable cardioverter-defibrillator infections. J Am Coll Cardiol.

[12] Polyzos KA, Konstantelias AA, Falagas ME (2015). Risk factors for cardiac implantable electronic device infection: a systematic review and meta-analysis. Europace.

[13] Cabell CH, Heidenreich PA, Chu VH, Moore CM, Stryjewski ME, Corey GR, Fowler VG Jr (2004). Increasing rates of cardiac device infections among Medicare beneficiaries: 1990-1999. Am Heart J.

[14] Voigt A, Shalaby A, Saba S (2010). Continued rise in rates of cardiovascular implantable electronic device infections in the United States: temporal trends and causative insights. Pacing Clin Electrophysiol.

[15] Vasilev S (2021). Strategies for prevention of surgical site infection in patients with CIED implantation: a literature review. Cureus.

[16] Sadeghi H, Alizadehdiz A, Fazelifar A, Emkanjoo Z, Haghjoo M (2018). New insights into predictors of cardiac implantable electronic device infection. Tex Heart Inst J.

[17] Prutkin JM, Reynolds MR, Bao H, Curtis JP, Al-Khatib SM, Aggarwal S, Uslan DZ (2014). Rates of and factors associated with infection in 200 909 Medicare implantable cardioverter-defibrillator implants: results from the National Cardiovascular Data Registry. Circulation.

[18] Tarakji KG, Ellis CR, Defaye P, Kennergren C (2016). Cardiac implantable electronic device infection in patients at risk. Arrhythm Electrophysiol Rev.

[19] Voigt A, Shalaby A, Saba S (2006). Rising rates of cardiac rhythm management device infections in the United States: 1996 through 2003. J Am Coll Cardiol.

[20] Geerlings SE, Hoepelman AI (1999). Immune dysfunction in patients with diabetes mellitus (DM). FEMS Immunol Med Microbiol.

[21] Hussein AA, Baghdy Y, Wazni OM (2016). Microbiology of cardiac implantable electronic device infections. JACC Clin Electrophysiol.

[22] Bloom H, Heeke B, Leon A, Mera F, Delurgio D, Beshai J, Langberg J (2006). Renal insufficiency and the risk of infection from pacemaker or defibrillator surgery. Pacing Clin Electrophysiol.

[23] Bongiorni MG, Tascini C, Tagliaferri E (2012). Microbiology of cardiac implantable electronic device infections. Europace.

[24] Tarakji KG, Wazni OM, Harb S, Hsu A, Saliba W, Wilkoff BL (2014). Risk factors for 1-year mortality among patients with cardiac implantable electronic device infection undergoing transvenous lead extraction: the impact of the infection type and the presence of vegetation on survival. Europace.

[25] Fukunaga M, Goya M, Nagashima M (2017). Identification of causative organism in cardiac implantable electronic device infections. J Cardiol.

[26] Arnold CJ, Chu VH (2018). Cardiovascular implantable electronic device infections. Infect Dis Clin North Am.

